# Two Novel Y-Type High Molecular Weight Glutenin Genes in Chinese Wheat Landraces of the Yangtze-River Region

**DOI:** 10.1371/journal.pone.0142348

**Published:** 2015-11-05

**Authors:** Yanchun Peng, Kan Yu, Yujuan Zhang, Shahidul Islam, Dongfa Sun, Wujun Ma

**Affiliations:** 1 College of Plant Sciences and Technology, Huazhong Agricultural University, Wuhan, 430070, China; 2 State Agriculture Biotechnology Centre, School of Veterinary & Life Sciences, Murdoch University, Perth, WA, 6150, Australia; 3 Australia Export Grains Innovation Centre (AEGIC), Perth, WA, 6150, Australia; 4 Hubei Collaborative Innovation Center for Grain Industry, Jingzhou, 434025, China; Huazhong University of Science & Technology(HUST), CHINA

## Abstract

High molecular weight glutenin subunits (HMW-GSs) are key determinants for the end-use quality of wheat. Chinese wheat landraces are an important resource for exploring novel HMW-GS genes to improve the wheat baking quality. Two novel *Glu-1Dy* HMW-GSs (designated as 1Dy12.6 and 1Dy12.7) were identified and cloned from two Chinese wheat landraces Huazhong830 and Luosimai. The 1Dy12.6 and 1Dy12.7 subunits were deposited as the NCBInr Acc. No KR262518, and KR262519, respectively. The full open reading frames (ORFs) of *1Dy12*.*6* and *1Dy12*.*7* were 2022 bp and 1977 bp, encoding for proteins of 673 and 658 amino acid residues, respectively. Each contains four typical primary regions of HMW-GSs (a signal peptide, N- and C-terminal regions, and a central repetitive region). Their deduced molecular masses (70,165 Da and 68,400 Da) were strikingly consistent with those identified by MALDI-TOF-MS (69,985Da and 68,407 Da). The 1Dy12.6 is the largest 1Dy glutenin subunits cloned in common wheat up to date, containing longer repetitive central domains than other 1Dy encoded proteins. In comparison with the most similar active 1Dy alleles previously reported, the newly discovered alleles contained a total of 20 SNPs and 3 indels. The secondary structure prediction indicated that 1Dy12.6 and 1Dy12.7 have similar proportion of α-helix, β-turn, and β-bend to those of 1Dy10 (X12929). The phylogenetic analysis illustrated that the x- and y-type subunits of glutenins were well separated, but both *1Dy12*.*6* and *1Dy12*.*7* were clustered with the other *Glu-1Dy* alleles. Our results revealed that the 1Dy12.6 and 1Dy12.7 subunit have potential to strengthen gluten polymer interactions, and are valuable genetic resources for wheat quality improvement.

## Introduction

Wheat (*Triticum aestivum* L.) is a significantly distinctive cereal crop by forming flour dough with visco-elastic properties that can be processed to produce a vast variety of foodstuffs such as steamed buns, cakes, biscuits, noodles, sour dough breads and pizzas. It is a staple food containing premium dietary fiber and vegetable protein of great nutritional value for human healthy diet. The viscous and elastic properties derived from two main protein groups, monomeric gliadins and polymeric glutenins, each containing various components of low and high molecular weight subunits [1]. A great progress has been achieved in understanding the structure, function, genetic expression, regulation and evolution of glutenin subunits in wheat and its related species [2–15]. High molecular weight glutenin subunits with relative molecular masses ranging from 60,000 Da to 90,000 Da are key constituents in their ability to form wheat dough strength, substantially influencing the end-use quality of wheat flour for bread-making [16–20]. In common wheat, they are encoded by *Glu-1* loci located on the long arms of chromosomes 1A, 1B and 1D [21]. Each locus contains two tightly linked genes encoding larger x-type and smaller y-type subunits with relative molecular masses in the 82,000–90,000 Da and 60,000–80,000 Da range, respectively [20, 21]. The y-type subunits generally exhibit relatively faster electrophoretic mobility on SDS-PAGE [20,21]. Both x- and y-type subunits possess similar primary structures, containing a signal peptide, a non-repetitive N-terminal region, a non-repetitive C-terminal region, and a long repetitive central region [22]. Differences in their molecular mass mainly result from the peptide length variation of their repetitive regions [23]. In the long repetitive central regions, three primary repeat units, tripeptides (GQQ), hexapeptides (PGQGQQ), and nonapeptides (GYYPTSLQQ) are identified [24]. Both x- and y-type subunits possess hexapeptide and nonapeptide unit, however the tripeptide units only exist in the x-type subunits [24]. Usually, seven and four cysteine residues conserved in y- and x-type subunits, respectively [24]. The y-type glutenin subunits possess more cysteine residues than x-type subunits, therefore are capable of forming more inter- and intra-molecular disulfide bonds, which aggregate HMW-GSs with each other and with LMW-GSs, resulting in improving dough quality [25].

In 1987, Payne et al. [16] demonstrated that allelic variation of the HMW-GSs composition in common wheat is associated with dough visco-elastic properties related to bread-making quality, which has stimulated great interests to identify and characterize novel HMW-GSs with different molecular structures [2–15]. Some HMW-GSs such as 1Ax1, 1Ax2*, 1Bx7^OE^, and 1Bx17 + 1By18 are found to possess positive effects on dough characteristics, while 1AxNull, 1Bx20, 1Bx6 + 1By8, 1Dx2 +1Dy12 have negative effects on gluten quality and bread-making quality [24,26–30]. Different HMW-GSs were endowed certain quality scores, and are widely applied as markers in wheat quality breeding programs to help selecting specific lines for different end-uses [31].

Sodium Dodecyl Sulfate Polyacrylamide Gel Electrophoresis (SDS-PAGE) has been employed widely for separation and identification of HMW and LMW subunits based on their different electrophoretic mobility [21,32–33]. However, some HMW-GSs possessing nearly identical relative molecular mass and electrophoretic mobility, such as 1Dx2 and 1Ax2*, 1Bx7 and 1Bx7*, 1Bx14 + 1By15 and 1Bx20, cannot be reliably distinguished from each other using SDS-PAGE [25,30,34]. Currently, a number of novel HMW-GSs have been found from common wheat and its relatives using matrix-assisted laser desorption/ionization time-of-flight mass spectrometry (MALDI-TOF-MS) technology, which represented a powerful high throughput and time saving tool for accurately and sensitively analyzing wheat glutenin subunits [35–41].

The HMW-GSs encoded by the *Glu-1D* locus are found to be responsible for major wheat dough quality variances, particularly elasticity and strength, and have been successfully employed in wheat quality improvement [42,43]. Up to now, only five active *Glu-1Dy* genes have been cloned and characterized from bread wheat [44]. Recently, we characterized the allelic variation at the *Glu-1* locus in 485 Chinese wheat landraces using MALDI-TOF-MS method, and identified several novel *Glu-1* subunits [41]. In the present research, we cloned two novel *Glu-1Dy* genes from Chinese wheat landraces. Their molecular characterizations were analyzed. The evolutionary biology of the *Glu-1* genes, and their potential value in common wheat processing quality were explored.

## Material and Methods

### 2.1 Plant Material and Field Trials

Two Chinese wheat landraces Huazhong830 and Luosimai containing novel candidate 1Dy subunits [20,41], originally collected from the Yangtze-River region, were used for cloning the novel HMW-GS genes. We confirmed that no permissions were needed for the original collection. The hexaploid wheat cv. Chinese spring (Null, 1Bx7 + 1By8, 1Dx2 + 1Dy12) was used as the control to compare the electrophoretic mobility of HMW-GSs on SDS-PAGE. The field trials acquired the approval of Huazhong Agricultural University, and were carried out on the experimental wheat farm of Huazhong Agricultural University, Wuhan (30°33′03″N 114°16′59″E), China. The land used belongs to Huazhong Agricultural University, which is not privately owned. The two landraces used were collected by professor Dongfa Sun in Wuhan (Huazhong830) and Yichang (Luosimai), China, and no any protected or endangered landraces were sampled in the field studies. The landraces were planted at the end of October in 2012, in three rows with 2 m in length and 20 cm between rows, and 12 plants in each row.

### 2.2 Matrix Assisted Laser Desorption/Ionization Time of Flight Mass Spectrometry (MALDI-TOF-MS) Analysis of HMW-GSs

HMW-GSs were extracted from seeds according to the protocol of Liu et al. with some modifications [20]. Seeds were crushed and extracted in 1.5 ml of 55% propanol-1-ol (v/v) for 1 hour, followed by incubation for 30 min at 65°C, vortexing for 5 min, and centrifugation for 5 min at 9,000 g. These steps were repeated three times to eliminate gliadin proportion. The HMW-GS present in the precipitation was treated with 55% propanol-1-ol, 0.08 M Tris-HCl solution containing 1% dithiothreitol (DTT), after which glutenin was precipitated in the 40% acetone.

MALDI-TOF-MS was done at the State Agriculture Biotechnology Centre, Murdoch University, Australia. The air-dried HMW-GS pellets were dissolved in 60 μl acetonitrile (ACN)/H_2_O (v/v, 50:50) with 0.05% v/v trifluoroacetic acid (TFA) for 1 h at room temperature. HMW-GS preparation was performed based on the dried droplet procedure [45], and sinapinic acid (SA) was employed as matrix. The matrix solution contains 10 mg SA in 1 mL 50% ACN/0.05% TFA (w/w). The prepared HMW-GS solution (total 60 μl) was mingled with SA solution at the ratio of 1:9 (v/v), and 2 μl of the glutenin–SA mixture was spotted onto a MALDI-TOF Voyager DE Pro 100 sample size plate, and dried at room temperature.

MALDI-TOF-MS was carried out on a Voyager DE-PRO TOF mass spectrometer (Applied Bio-systems, Foster City, CA, USA) equipped with a 337 nm nitrogen laser. The experiments were performed on a positive linear ion mode with the following parameters: mass range 50–100 kDa, laser intensity 2500, acceleration voltage 25 kV, delay time 850 ns, grid voltage 92%, guide wire 0.3%, bin size 20 ns and input bandwidth 20 MHz. Spectra were averaged from 50 laser shots in order to improve the signal-to-noise level.

### 2.3 SDS-PAGE Analysis of HMW-GSs

The extraction of HMW-GSs and their SDS-PAGE analysis were performed based on the methods of Liu et al. [20]. Single crushed seed (20mg) was suspended in 1 ml of 62.5 mM Tris-HCl solution (pH 6.8), containing 0.002% (w/v) bromophenol blue, 1.5% (v/v) dithiothreitol, 2% (w/v) SDS and 10% (v/v) glycerol for 3 min with continuous vortexing and followed by incubation for 5 min at 100°C. After centrifugation at 18,000 g for 3 min, the supernatant was used for SDS-PAGE analysis. HMW-GSs were separated using 10% SDS-PAGE with Tris-Glycine-SDS running buffer as described previously [20].

### 2.4 DNA Extraction and PCR Amplification

Genomic DNA was extracted from dry seeds using a CTAB method [20]. Two PCR primers were designed based on the sequences of the published HMW-GS genes to amplify the complete ORF (P1: 5’-ATGGCTAAGCGGTTGGTCCT-3’, and P2: 5’-TCACTGGCTAGCCGACAATG-3’) [20]. PCR was accomplished in a 50 μL reaction mixture, containing 1× GC buffer Ⅰ, 0.3 μM of each primer, 200 μM of each dNTP, 2.5 U LA *Taq* polymerase and 500 ng of genomic DNA. The PCR amplification condition included an initial step at 94°C for 3 min, followed by 28 cycles of 94°C denaturation for 1 min, 62°C annealing for 1 min and 72°C extension for 2 min, and a final step at 72°C for 10 min. The PCR product was separated on 1.5% agarose gel. Electrophoresis was carried out at 60 V for 40 min at room temperature.

### 2.5 Molecular Cloning and Sequencing

The target DNA fragments were purified from agarose gel using the Gel Extraction Kit (Omega) and then ligated into the pGEM-T easy vector (Promega, USA). The ligation mixture was transformed into *Escherichia coli* JM109 competent cells. The full length sequence of *Glu-1Dy* ORF was obtained by primer walking. The three positive clones for each 1Dy gene were sequenced commercially by the State Agriculture Biotechnology Centre, Perth, Australia.

The gene sequences were assembled, and compared among different HMW-GS genes using DNAMAN Version 6.0.3.99 software. The translation of nucleotide sequences was carried out using DNASTAR software suite (Version 7.1.0). The SSpro8 (http://scratch.proteomics.ics.uci.edu/) was performed to predict the secondary structure of deduced amino acid sequences.

In order to analyze phylogenetic relationships among HMW-GS gene alleles, a multiple alignment was carried out with the ClustalW program. A phylogenetic tree was constructed using neighbour-joining (NJ) method in MEGA6.06 [46] based on the deduced amino acid sequences from the novel cloned two 1Dy and other *Glu-1* genes from different genomes, including 1Ax1 (X61009), 1Ax2* (M22208), 1Ax2*^B^ (EF055262), 1Ax1.1 (JN172932), 1Bx7 (X13927), 1Bx7^OE^ (DQ119142), 1Bx13 (EF540764), 1Bx14 (AY367771), 1Bx20 (AJ437000), 1Dx2 (X03346), 1Dx5 (X12928), 1By8 (AY245797), 1By9 (X61026), 1By15 (EU137874), 1By16 (EF540765), 1Dy10 (X12929), 1Dy10.1 (AY695379), 1Dy12.3 (EF472958), 1Sy18* (JQ680980), Sy9* (HQ380223), 1Dy12.4*t (KC196061), 1Dy12.1 (AY248704), 1Dy10.5t (FJ499503), and 1Dy12 (X03041). The number of bootstrap replicates was 500 [47].

## Results

### 3.1 Characterization of 1Dy12.6 and 1Dy12.7 Subunits by MALDI-TOF-MS and SDS-PAGE

**Fig 1:**
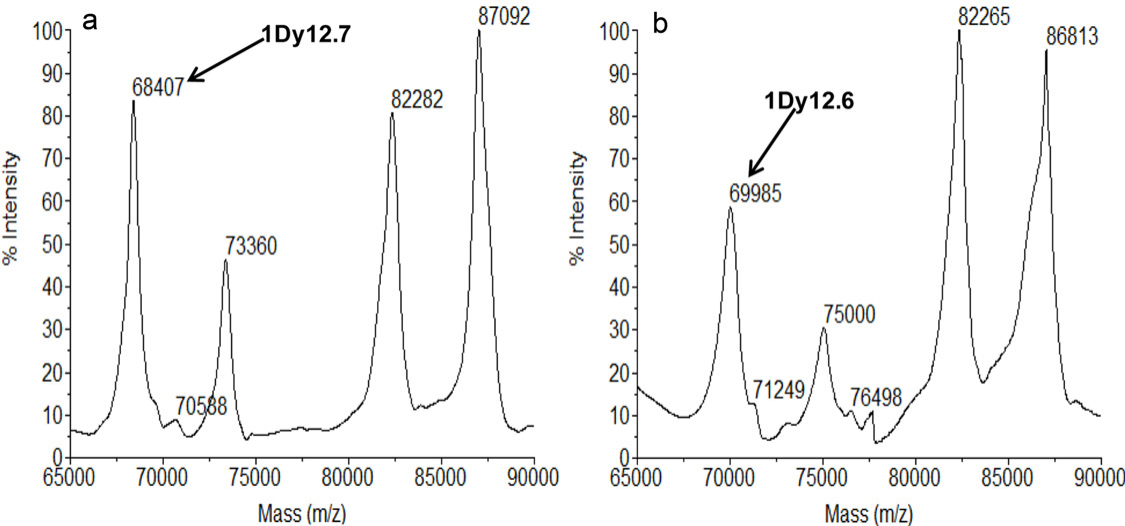
MALDI-TOF analysis of HMW-GS components in Chinese wheat landraces. a. Luosimai; b. Huazhong830.

**Fig 2:**
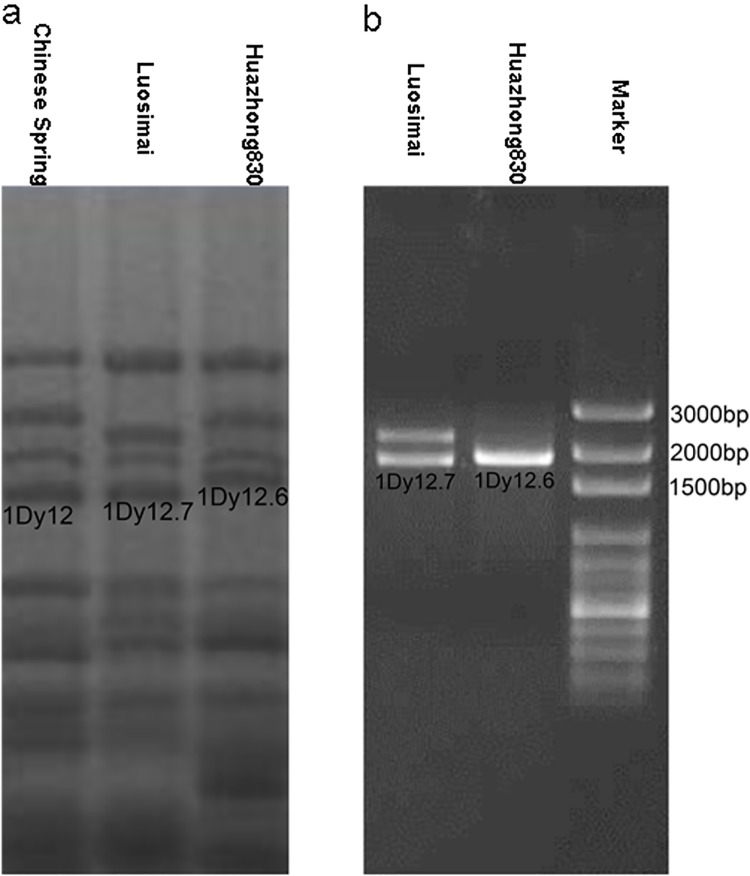
a. SDS-PAGE analysis of HMW-GS components in two Chinese wheat landraces. b. PCR amplification of the 1Dy genes.

HMW-GS profiles of the two Chinese wheat landraces were obtained by MALDI-TOF-MS (Fig 1). Both Huazhong830 and Luosimai have four HMW-GS peaks with relative molecular masses ranging from 65,000 to 90,000 Da. In comparison with the study of Liu et al. [20], two novel candidate 1Dy subunits with relative molecular mass of 69,985 Da (designated as 1Dy12.6) and 68407 Da (designated as 1Dy12.7) were larger than or close to that of 1Dy12 (68300 Da, GenBank: X03041), one of common HMW-GS reported previously [20]. In order to further characterize the two 1Dy subunits, SDS-PAGE method was carried out to separate HMW-GS composition of Huazhong830 and Luosimai. The SDS-PAGE analysis showed that 1Dy12.7 possessed similar electrophoretic mobility to 1Dy12 in Chinese Spring; the mobility of subunit 1Dy12.6 from Huazhong830 was slower than that of the subunit 1Dy12 from Chinese Spring (Fig 2A). The MALDI-TOF-MS profiles were consistent with the electrophoretic mobility order of SDS-PAGE results.

### 3.2 Molecular Cloning and Characterization of the *1Dy12*.*6* and *1Dy12*.*7* genes

Since HMW-GS genes do not contain intron [24–25], the wheat genomic DNA can be used as a template to amplify the ORF region. A single amplified fragment about 2.0 kb in Huazhong830 was amplified using primers P1 and P2 (Fig 2B). The amplified PCR products from Luosimai consisted of two different fragments in size, about 2.2 kb and 2.0 kb, respectively (Fig 2B). The size of about 2.0 kb in length from the two Chinese wheat landraces, designated as *1Dy12*.*6* and *1Dy12*.*7* genes, respectively, were close to the size of 1Dy ORFs previously published. The length of their nucleotide sequences were 2022 bp and 1977 bp, containing 673 and 658 amino acid residues, respectively. Their deduced mature protein relative molecular masses (70,165 Da and 68,400 Da) were consistent with those identified by MALDI-TOF-MS (69,985 Da and 68,407 Da). The nucleotide and deduced amino acid sequences of the two novel *Glu-D1-2* genes have been deposited in the GenBank with the accession number KR262518 for *1Dy12*.*6* and KR262519 for *1Dy12*.*7*. To our knowledge, *1Dy12*.*6* was the largest active *Glu-1Dy* gene cloned up to date [44].

The deduced amino acid sequences of both 1Dy12.6 and 1Dy12.7 showed typical similar primary structural characteristics for HMW-GSs, which include a signal peptide of 21 amino acids removed from mature protein during the protein transportation to endoplasmic reticulum, followed by a non-repetitive conserved N-terminal region with 104 amino acids in both subunits, a long repetitive central domain of 506 and 491 residues for 1Dy12.6 and 1Dy12.7, respectively, and a conserved C-terminal region with 42 amino acids in both subunits. Both 1Dy12.6 and 1Dy12.7 have seven cysteine residues at the conserved positions, five in the N-terminal region, one in the central repetitive region close to the C-terminus and one in the C-terminal region. The central repetitive region of 1Dy12.6 and 1Dy12.7 is mainly composed of repeats of hexapeptide (consensus PGQGQQ) and nonapeptide (consensus GYYPTSLQQ) motifs, but without tripeptide (consensus GQQ). There are 48 hexapeptide and 20 nonapeptide repeat units in 1Dy12.6 subunit, in which hexapeptide units are contiguous or separated by other repeat motifs, and all nonapeptide units are separated by a hexapeptide unit or a PGQQ tetrapeptide. A total of 48 hexapeptide and 19 nonapeptide repeat units in 1Dy12.7 subunit were identified, hexapeptide motifs present in tandem or are separated by nonapeptide repeat units, and all nonapeptide units are separated by either a hexapeptide unit or a PGQQ tetrapeptide.

### 3.3 Comparison of *1Dy12*.*6* and *1Dy12*.*7* with Other *Glu-1-2* Alleles

Sequences comparison revealed that gene sequences of *1Dy12*.*6* and *1Dy12*.*7* were homologous to the 1Dy type (Table 1). The sequences of both the *1Dy12*.*6* and *1Dy12*.*7* display higher identities (from 90.52% to 99.70%) with 13 1Dy alleles than with other 13 y-type HMW-GS genes on A and B genomes (from 80.08% to 91.60%). In comparison with the 13 1Dy alleles retrieved from the GenBank, the sequence of *1Dy12*.*6* is similar to the sequence EU266533 from Slovak wheat variety ‘Trebišovská 76’, with identity of 99.70%, and *1Dy12*.*7* is similar to the sequence JF736016 from wheat cultivar Shinchunaga (97.44%) [44]. The length of both *1Dy12*.*6* and EU266533 sequences is 2022 bp, and longer than other sequences of 1Dy allele (Table 1) [44].

**Table 1:** Identities of sequences among the *1Dy12*.*6*, *1Dy12*.*7* and 26 other y-type HMW-GS genes from *Triticum aestivum*.

HMW-GS alleles	GenBank accession	Genome	Gene Expression	The length of ORF (bp)	Deduced AA length	Deduced relative molecular mass (Da)	Identities of 1Dy12.6	Identities of 1Dy12.7
1Dy12*	EU266533	AABBDD	inactive	2022	673	70201	99.70%	94.23%
1Dy12	JF736016	AABBDD	active	1977	658	68528	97.63%	97.44%
1Dy12	BK006459	AABBDD	active	1977	658	68528	97.58%	97.39%
1Dy12	AY486484	AABBDD	active	1977	658	68526	97.43%	97.24%
1Dy12	X03041	AABBDD	active	1983	660	68713	97.29%	97.05%
1Dy12.2*	FJ226583	AABBDD	active	1977	658	68523	96.84%	97.19%
1Dy12.3	EF472958	AABBDD	active	1959	652	67884	96.54%	96.39%
1Dy10.1	AY695379	AABBDD	active	1968	655	68195	96.39%	96.49%
1Dy12*	EU495302	AABBDD	active	1977	658	68513	96.14%	96.14%
1Dy11	EU528008	AABBDD	active	1914	637	66224	94.52%	90.98%
1Dy10	X12929	AABBDD	active	1947	648	67475	94.31%	90.67%
1Dy10	AB281268	AABBDD	active	1947	648	67547	94.26%	90.62%
1Dy10	EU287437	AABBDD	active	1947	648	67602	94.16%	90.52%
1By15*	KJ579440	AABBDD	active	2109	702	73201	91.60%	89.20%
1By8.1	HQ731654	AABBDD	inactive	2155	0	0	90.19%	90.01%
1By9	X61026	AABBDD	active	2118	705	73517	90.08%	82.78%
1By15	EU137874	AABBDD	active	2154	718	75150	88.22%	87.87%
1By15	KF733215	AABBDD	active	2154	718	74738	88.09%	87.94%
1By16	EF540765	AABBDD	active	2217	739	77283	87.57%	84.53%
1By15	DQ086215	AABBDD	active	2172	724	75735	87.16%	83.88%
1By8	JF736014	AABBDD	active	2163	720	75131	86.52%	83.94%
1By8	KF430649	AABBDD	active	2163	720	75187	86.46%	83.88%
1By8xym7	KF855989	AABBDD	active	2163	720	75131	86.34%	83.77%
1Ay	JF736012	AABBDD	inactive	1752	0	0	84.09%	80.76%
1Ay	X03042	AABBDD	inactive	1809	0	0	81.22%	81.03%
1Ay	KC545955	AABBDD	inactive	1791	0	0	80.42%	80.08%

### 3.4 Single Nucleotide Polymorphisms (SNPs) and Indels Identification

The ORF of *1Dy12*.*6* and *1Dy12*.*7* sequences was compared with that of the 1Dy active allele. A total of 20 SNPs and 3 Indels were detected at different positions along the sequences (Tables 2 and 3, S1 and S2 Figs). The 13 SNPs are transitions (A-G and T-C accounting for 65%) and 7 are transversions (A-C, A-T, and G-T accounting for 35%), which lead to 13 non-synonymous mutations, resulting in amino acid residue substitutions. In comparison with JF736016, a 45 bp insertion at the position of 958–1002 in the *1Dy12*.*6* was found, encoding a typical tandem nonapeptide and hexapeptide repetitive units (GHYPASQQQPGQGQQ). Only three SNPs between *1Dy12*.*6* and JF736016 were found at positions 368 (T→C, isoleucine→threonine), 1469 (G→A, arginine→glutamine), and 1687 (A→C, threonine→proline). Compared with JF736016, a 18 bp deletion at the position 540–541 coding a hexapeptide unit (QIGKGK) and a 18 bp insertion at the position from 700 to 717 encoding a hexapeptide unit (RQIGQG) were identified in the *1Dy12*.*7*. Seventeen SNPs between JF7360166 and *1Dy12*.*7* were detected. Of which, ten were non-synonymous mutation at position 482 (T→C, leucine→proline), position 500 (A→G, glutamic acid→tryptophan), position 522 (T→G, histidine→glutamine), position 526 (T→C, serine→proline), position 556 (T→A, serine→threonine), position 571 (T→C, serine→proline), position 634 (C→A, proline→threonine), position 656 (G→T, glycine→valine), position 692 (G→A, glycine→glutamic acid), and position 1001 (G→A, arginine→glutamine).

**Table 2:** SNPs and Indels analysis of *1Dy12*.*6* gene compared with its most similar active allele JF736016.

Gene	368	958–1002	1469	1687
1Dy12.6	T(I)	Indel1	G(R)	A(T)
JF736016	C(T)	***(*)	A(Q)	C(P)

Note: Indel1

GGGCCCAGCTTCTCAGCAGCAGCCAGGACAAGGGCAACAA (GHYPASQQQPGQGQQ)

The Indels were marked by the asterisks “*”. The letters and “*” in parentheses were the substitutions and deletions of amino acids derived from the corresponding SNPs and Indels, respectively.

**Table 3:** SNPs and Indels analysis of *1Dy12*.*7* compared with its most similar active allele JF736016.

Gene	465	482	483	499	500	522	526	537	540–541	556	564	571	634	656	666	692	700–717	1001	1227
1Dy12.7	A	T(L)	G	G	A(E)	T(H)	T(S)	A	***(*)	T(S)	A	T(S)	C(P)	G(G)	G	G(G)	Indel3	G(R)	G
JF736016	T	C(P)	A	T	G(W)	G(Q)	C(P)	G	Indel2	A(T)	G	C(P)	A(T)	T(V)	A	A(E)	***(*)	A(Q)	A

Note: Indel2, CAGATAGGAAAAGGGAAA (QIGKGK); Indel3, CGGCAAATAGGACAAGGG (RQIGQG)

The Indels were marked by the asterisks “*”. The letters and “*” in parentheses were the substitutions and deletions of amino acids derived from the corresponding SNPs and Indels, respectively.

### 3.5 Secondary Structure of 1Dy12.6, 1Dy12.7, 1Dy10 and 1Dy12

The secondary structures of the 1Dy12.6, 1Dy12.7, 1Dy10 (associated with good bread-making quality) and 1Dy12 (associated with poor bread-making quality) subunits were analyzed using SSpro8 system and the results were given in Table 4.

**Table 4:** The secondary structure prediction of four HMW-GSs.

HMW-GS	Motifs	Content (%)	Total (No.)	NT Content (%)	CR Content (%)	CT Content (%)	NT (No.)	CR (No.)	CT (No.)
1Dy12.6	alpha-helix	5.21	2	5.21	0	0	2	0	0
	beta-turn	7.67	43	1.23	6.13	0.31	6	36	1
	beta-bend	0.15	1	0	0	0.15	0	0	1
	the rest	85.89	47	9.2	71.47	5.21	8.5	36	2.5
	beta-strand	1.07	2	0.31	0	0.77	1	0	1
	3-10-helix	0	0	0	0	0	0	0	0
	pi-helix	0	0	0	0	0	0	0	0
	beta-bridge	0	0	0	0	0	0	0	0
1Dy12.7	alpha-helix	5.34	2	4.4	0	0.94	1	0	1
	beta-turn	8.01	47	1.57	6.28	0.16	8	38	1
	beta-bend	0.31	2	0.31	0	0	2	0	0
	the rest	86.34	50	10.04	70.81	5.49	9.5	38	2.5
	beta-strand	0	0	0	0	0	0	0	0
	3-10-helix	0	0	0	0	0	0	0	0
	pi-helix	0	0	0	0	0	0	0	0
	beta-bridge	0	0	0	0	0	0	0	0
1Dy10	alpha-helix	5.51	3	5.51	0	0	3	0	0
	beta-turn	8.59	39	1.3	6.32	0.97	6	36	5
	beta-bend	0.32	2	0.16	0	0.16	1	0	1
	the rest	85.25	51	9.89	70.02	5.35	7.5	36	7.5
	beta-strand	0.32	1	0	0	0.32	0	0	1
	3-10-helix	0	0	0	0	0	0	0	0
	pi-helix	0	0	0	0	0	0	0	0
	beta-bridge	0	0	0	0	0	0	0	0
1Dy12	alpha-helix	4.38	1	4.38	0	0	1	0	0
	beta-turn	7.82	46	1.56	5.95	0.31	8	36	2
	beta-bend	0.16	1	0	0	0.16	0	0	1
	the rest	87.32	49	10.02	71.21	6.1	10	36	3.5
	beta-strand	0.31	1	0.31	0	0	1	0	0
	3-10-helix	0	0	0	0	0	0	0	0
	pi-helix	0	0	0	0	0	0	0	0
	beta-bridge	0	0	0	0	0	0	0	0

Note: NT: N-terminal region; CT: C-terminal region; CR: central repetitive domain.

As shown in Table 4, 1Dy12.6 subunit possesses four kinds of secondary structure motifs, including α-helix (5.21%), β-turn (7.67%), β-bend (0.15%) and β-strand (1.07%), and the rest (85.89%). The β-turns are distributed in each of the three domains, mainly in central repetitive domain (6.13%), two α-helices only exist in the N-terminal, one β-bend exists in the C-terminal domain, and two β-strand are distributed in the N-terminal and C-terminal domain. 1Dy12.7 subunit has three kinds of secondary structure motifs, viz., 2 α-helices, 47 β-turns and 2 β-bends, and 50 the rests. In comparison with the 1Dy12, high proportion of α-helix is identified in the 1Dy12.6, 1Dy12.7 and 1Dy10. The content of β-turns in the four subunits is similar (approximately 8%). None of 3-10-helix, pi-helix, β-bridge were present in the four subunits.

### 3.6 Phylogenetic Analysis

**Fig 3:**
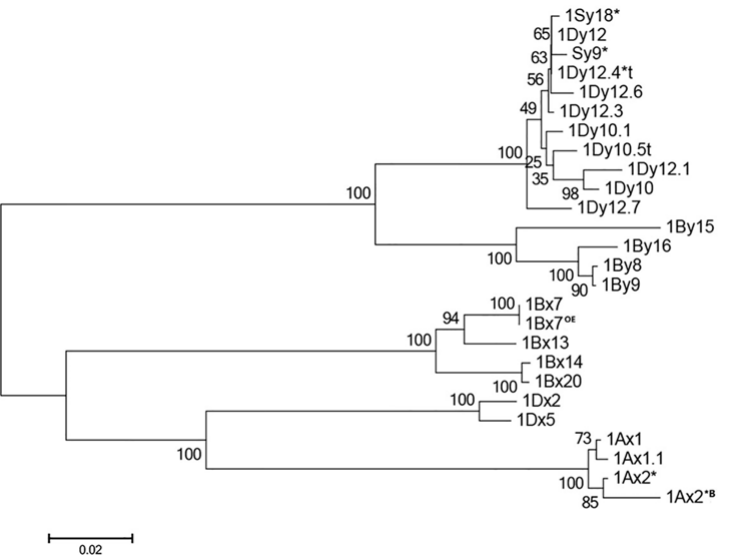
The phylogenetic tree of 26 HMW-GSs based on deduced amino acid sequences. *1Dy10*.*5t*, *1Dy12*.*1* and *1Dy12*.4*t from *Aegilops tauschii*; Sy9* and 1Sy18* from *Aegilops speltoides*; and 21 genes from *Triticum aestivum*.

To investigate the evolutionary relationships among the two novel 1Dy genes and other representative HMW-GS alleles, a phylogenetic tree was constructed based on the deduced amino acid sequences of 26 *Glu-1* alleles, which included the novel *1Dy12*.*6* and *1Dy12*.*7* genes cloned in this research and other 24 HMW-GS genes retrieved from the GenBank. As shown in Fig 3, the phylogenetic tree clearly separated the 26 *Glu-1* sequences into two groups, the x-type alleles of *Glu-1-1* in one group and the y-type *Glu-1-2* alleles in another group. Within the x-type allele group, the alleles from same genome were grouped together, clearly formed A, B and D genome subgroups. The x-type genes on A genome was sister to the genes on the D genomes. The y-type alleles were divided into two subclades, one comprised of the alleles from B genome and another comprised of the alleles from S and D genome. The *1Dy12*.*6* gene was closely related with *1Dy12*, *1Dy12*.4*t, Sy9* and 1Sy18*, whereas *1Dy12*.*7* gene was separated from Sy and other 1Dy alleles.

## Discussion

Wheat is an unique cereal crop due to the elasticity and viscosity conferred by wheat dough. Based on these special rheological properties, wheat flour has been used to produce a vast variety of foods, making it one of the staple foods for human diet. The viscous and elastic properties are mainly derived from two main storage protein groups, monomeric gliadins and polymeric glutenins in the endosperm, notably HMW-GSs [48]. In our study, two novel HMW-GS genes (*1Dy12*.*6* and *1Dy12*.*7*) from Chinese wheat landraces have been cloned and characterized.

The deduced protein sequences of two novel genes possess the typical feature of y-type glutenin subunit. Comparative sequence analysis showed that *1Dy12*.*6* and *1Dy12*.*7* genes are more similar to the glutenin genes on D genome. The phylogenetic tree showed that they obviously separated from the 1Dy subgroup, confirming that they belong to y-type subunit.

Previous studies proposed that a long repetitive central domain has a positive influence on dough visco-elastic properties since they can form more polymer–polymer interactions conferring elasticity to the protein polymers [48–51]. Molecular characterization revealed that 1Dy12.6 contains 673 amino acids with a 15 amino acid residue insertion in the central repetitive domain (one hexapeptide and one nonapeptide) compared with JF736016, which might be due to slip mismatching or unequal crossover event. The sequence of *1Dy12*.*6* is longer than other active 1Dy allele, and is the longest active 1Dy allele reported up to date [17]. The 1Dy12.6 subunit with a relative molecular mass of 69,985 Da possesses longer central repetitive domain than other 1Dy subunit, which may play a positive role in improving dough visco-elastic properties.

Some research have revealed that a set of intrachain and interchain disulphide bonds existed in HMW-GSs, including one interchain disulphide bond within the N-terminal region of an x-type subunit, two disulphide bonds between the N-terminal region of y-type subunits, one disulphide bond between LMW-GSs and y-type subunits, and one disulphide bond linking the x-type and y-type HMW-GSs [29,52–53]. These bonds form giant polymers [29,52–53]. The distribution and number of cysteine residues in HMW-GSs were believed to be important factors influencing wheat bread making quality [29,53]. In comparison with 1Bx7 subunit, two cysteine residues present in the N-terminal domain of 1Bx20 subunit, which were believed to be responsible for its negative effect on dough strength, and decrease the number of intrachain bonds and change the disulphide bond linking patterns in the wheat glutenin polymers [29]. However, an extra cysteine residue in the central repetitive domain of 1Dx5 subunit was considered to be responsible for its positive effect on quality property [53]. In this study, both novel genes displayed the same number of cysteine residues and distribution as other typical y-type HMW-GSs do, containing five in the N-terminal domain, one in the central domain close to the C-terminus and one in the C-terminal domain, which may be important for forming the normal gluten polymers.

The positive relationship between glutamine content in HMW-GS and its effect on dough strength has been revealed previously [49,54]. Theoretically, the high glutamine residue content helps form more hydrogen bonds in protein–protein interactions (“loop and train” model hypothesis) and maintain the structure of glutenin polymers [49,54–56]. In this study, although 1Dy12.6, 1Dy12.7, 1Dy10 and 1Dy12 possess different relative molecular masses and amino acids length, they contained very similar number and content of glutamine residues, which may form similar number of hydrogen bonds in polymers.

In this study, the secondary structures of 1Dy12.6 and 1Dy12.7 were predicted (Fig 3). The results showed that 1Dy12.6 and 1Dy12.7 subunits have similar content of α-helix, β-turn, and β-bend compared with the secondary structure of 1Dy10, which might be beneficial to the formation of wheat gluten polymers [28,49,57–58].

As HMW-GSs play a significant role in wheat bread making quality, the two novel genes can be used as special genetic resources for wheat quality breeding. In the future, it is important to explore the two novel HMW-GS alleles affecting end use value. We are under the way to create transgenic wheat that specifically expresses these novel 1Dy alleles in the endosperm to verify the function of these two novel HMW-GS alleles.

## Supporting Information

S1 Fig Alignment of the predicted primary structure of 1Dy12.6 and 1DyJF736016 HMW-GSs. The dots indicate the deletion of amino acids relative to the 1Dy12.6 sequence. Substitutions are indicated by white and nattier blue background colors.
(TIF)

S2 Fig Alignment of the predicted primary structure of 1Dy12.7 and 1DyJF736016 HMW-GSs. The dots indicate the deletion of amino acids relative to the other sequence. Substitutions are indicated by white and nattier blue background colors.
(TIF)

## Abbreviations

HMW-GS: high molecular weight glutenin subunit
MALDI-TOF-MS: matrix-assisted laser desorption/ionization time of flight mass spectrometry
SDS-PAGE: sodium dodecyl sulfate polyacrylamide gel electrophoresis
PCR: polymerase chain reaction
