# Supplementary figures and images for: Two Novel Y-Type High Molecular Weight Glutenin Genes in Chinese Wheat Landraces of the Yangtze-River Region
Source: PLoS One. 2015 Nov 5;10(11):e0142348. doi: 10.1371/journal.pone.0142348 (PMC4635010; doi:10.1371/journal.pone.0142348)

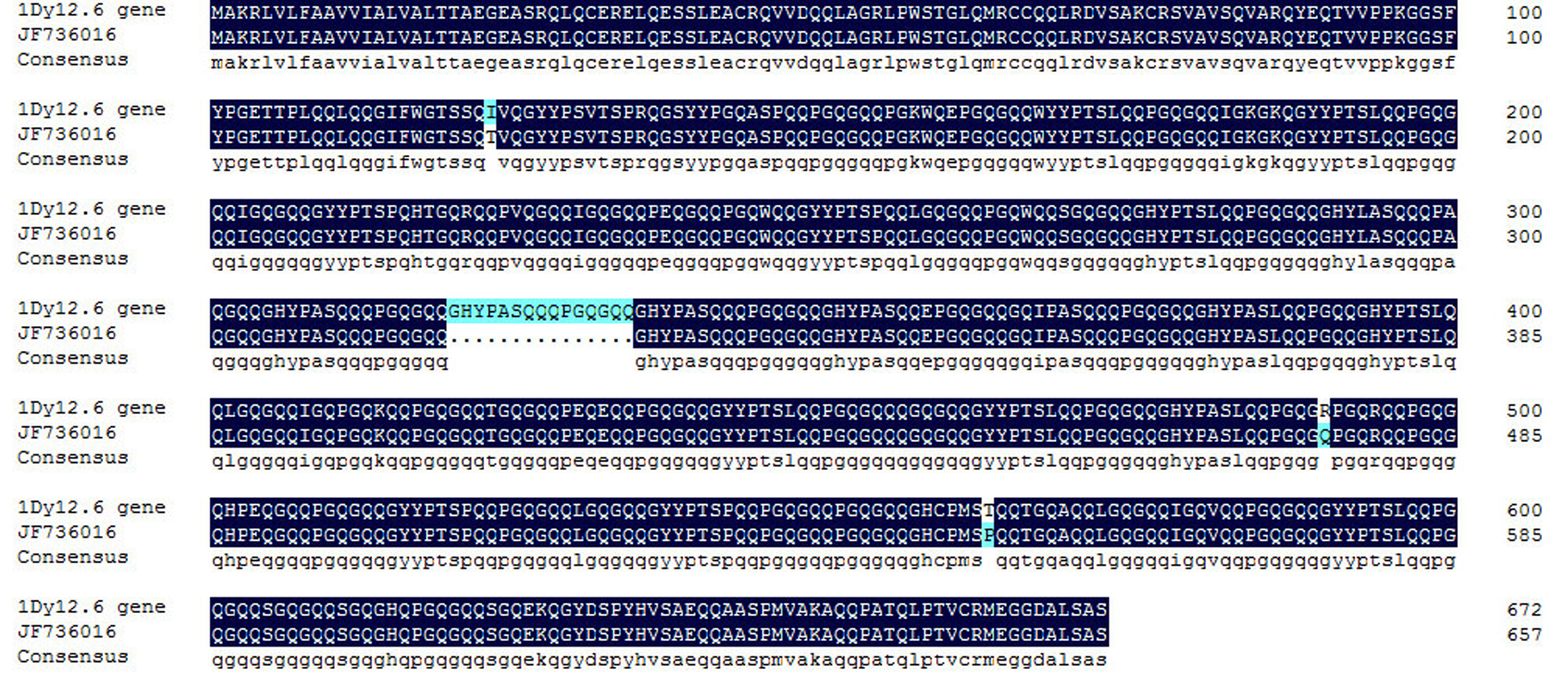

Supplement: S1 Fig — The dots indicate the deletion of amino acids relative to the 1Dy12.6 sequence. Substitutions are indicated by white and nattier blue background colors. (TIF) [file pone.0142348.s001.tif]

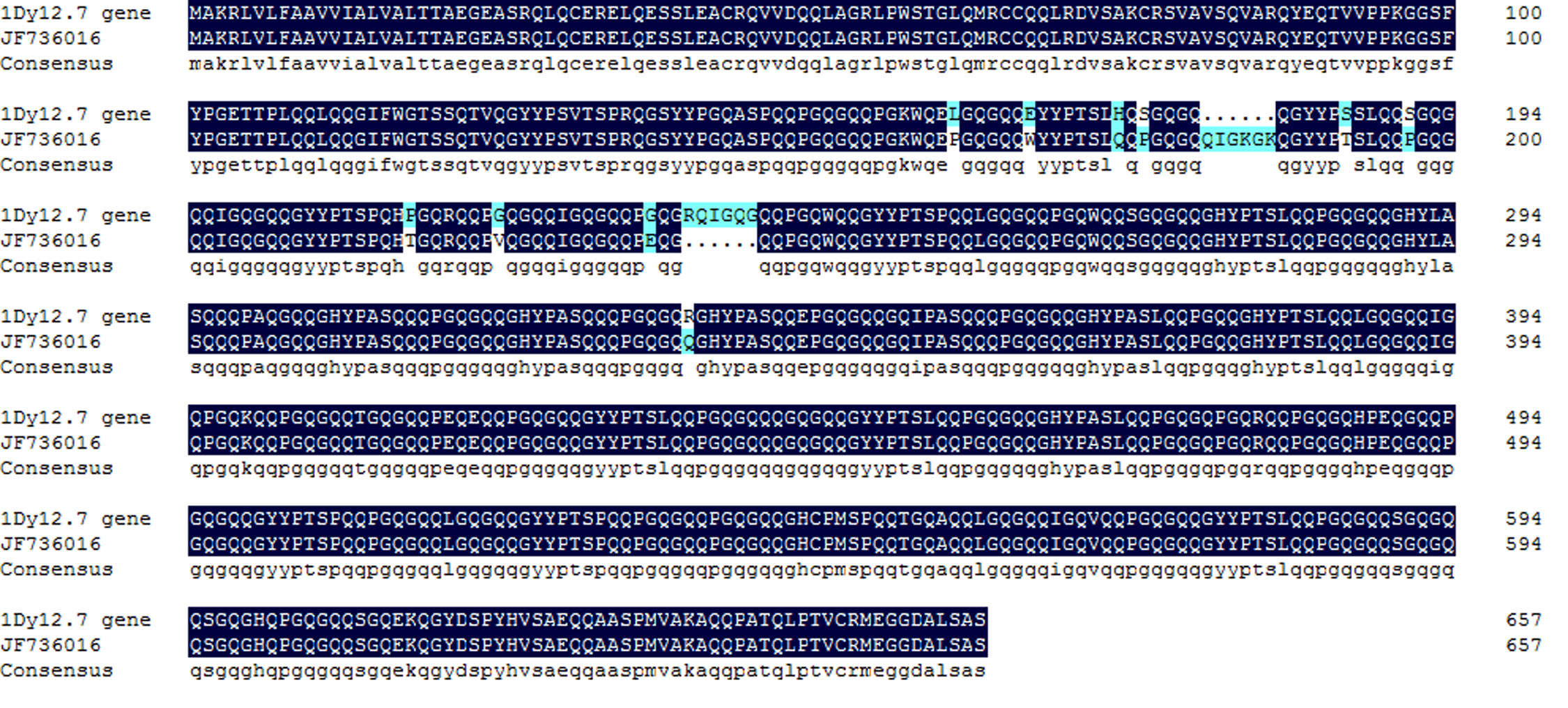

Supplement: S2 Fig — The dots indicate the deletion of amino acids relative to the other sequence. Substitutions are indicated by white and nattier blue background colors. (TIF) [file pone.0142348.s002.tif]
